# Endoscopy-assisted high cervical anterolateral retropharyngeal approach to clivus: a cadaveric study

**DOI:** 10.3389/fsurg.2024.1397729

**Published:** 2024-07-22

**Authors:** Caner Cicek, Ömer Orhun, Orhun Mete Cevik, Lala Rzayeva, Mustafa Güdük, Murat İmre Usseli, Mehmet Emin Aksoy, Erhan Emel, M. Necmettin Pamir, Baran Bozkurt

**Affiliations:** ^1^ Department of Neurosurgery, Zonguldak State Hospital, Zonguldak, Türkiye; ^2^ Department of Neurosurgery, Cerrahpasa Medical Faculty, Istanbul University-Cerrahpasa, Istanbul, Türkiye; ^3^ Department of Neurosurgery, School of Medicine, Acibadem Mehmet Ali Aydınlar University, Istanbul, Türkiye; ^4^ School of Medicine, Acıbadem Mehmet Ali Aydinlar University, Istanbul, Türkiye; ^5^ Department of Neurosurgery, Bakirkoy Teaching and Research Hospital for Psychiatric and Nervous Diseases, University of Health Sciences, Istanbul, Türkiye

**Keywords:** anterior clivectomy, endoscopic assisted approach, craniovertebral junction, transcervical approach, neuroanatomy, cadaveric study

## Abstract

**Introduction:**

The surgical management of pathologies involving the clivus and craniocervical junction has always been considered a complex procedure because of the deeply located surgical targets and the surrounding complex neural and vascular anatomical structures. The most commonly used approaches to reach this area are the transnasal, transoral, and transcervical approaches.

**Material and Methods:**

This approach was performed unilaterally on five cadaver heads and bilaterally on one cadaver head.

**Results:**

We described a modified endoscope-assisted high cervical anterolateral retropharyngeal approach in which each stage of the procedure was demonstrated on human cadavers in a step-by-step manner using endoscopic camera views. This approach was broken down into nine steps. The neurovascular structures encountered at each step and their relationships with each other are demonstrated.

**Discussion:**

The advantages and disadvantages of our modified approach were compared to the conventional transcervical, transoral, and endoscopic endonasal approaches.

## 1 Introduction

The most common tumors of the lower clival region are chordomas and chondrosarcomas, followed by other rare tumor types such as plasmacytomas and pituitary adenomas, which have been described either with extra- or intra-dural localization (1). The surgical management of pathologies involving the clivus and craniocervical junction has always been considered a complex procedure because of the deeply located surgical site and the surrounding complex neural and vascular anatomical structures. Many approaches have been proposed in the literature with variable advantages and disadvantages (2). The routes of these approaches utilize endonasal, transoral, and transcervical corridors while using a surgical endoscope or microscope (3).

In 1957, Southwick and Robinson described the anterior retropharyngeal pre-vascular approach to the upper cervical spine (4). In 1986, Lesoin et al. (5), and in 1987 McAfee et al., modified and popularized this approach and provided extended access to the clivus and the upper three cervical vertebrae (6). Stevenson et al. reported the first study describing a high anterolateral retropharyngeal pre-vascular approach for the management of a clival chordoma (7). In 2011, Russo et al. performed 20 mm × 20 mm minimal anterior clivectomy with the high anterior cervical approach. It also showed the anatomical structures exposed by minimal anterior clivectomy and demonstrated that the cranial nerves (CN) VI, IX, X, and XI; the anterior inferior cerebellar artery (AICA); and the basilar artery (BA) can be seen in any cadaver with minimal anterior clivectomy (8). With the widespread use of endoscopes in neurosurgery, it has been shown that the endoscope can also be used in the anterior cervical approach (9, 10).

In this cadaveric study, we described a modified endoscope-assisted high cervical anterolateral retropharyngeal approach (HCALR) in which each stage of the dissection was demonstrated step by step with endoscopic camera views. Comparisons of previously described approaches that emphasize advantages and disadvantages have also been discussed.

## 2 Material and methods

Six adult cadaveric specimens with colored silicone-injected vasculature were used. While performing the high cervical anterolateral retropharyngeal approach, each step of the dissection was documented using a rigid endoscope (Karl Storz®, STORZ, Germany) 4 mm in diameter, 18 cm in length, with 0° lenses, and a Canon EOS 5D Mark II (Canon Incorporated, Tokyo, Japan) camera. This approach was performed bilaterally in one cadaver and unilaterally in the remaining cadavers. C-arm fluoroscopy was used to confirm localization. The dissections were performed at the Center of Advanced Simulation and Education Neuroanatomy Laboratory, Acıbadem MAA University in İstanbul, Turkey.

## 3 Results

### 3.1 Surgical procedure

#### 3.1.1 Surgical approach is broken down into nine steps and described accordingly

**Figure 1:**
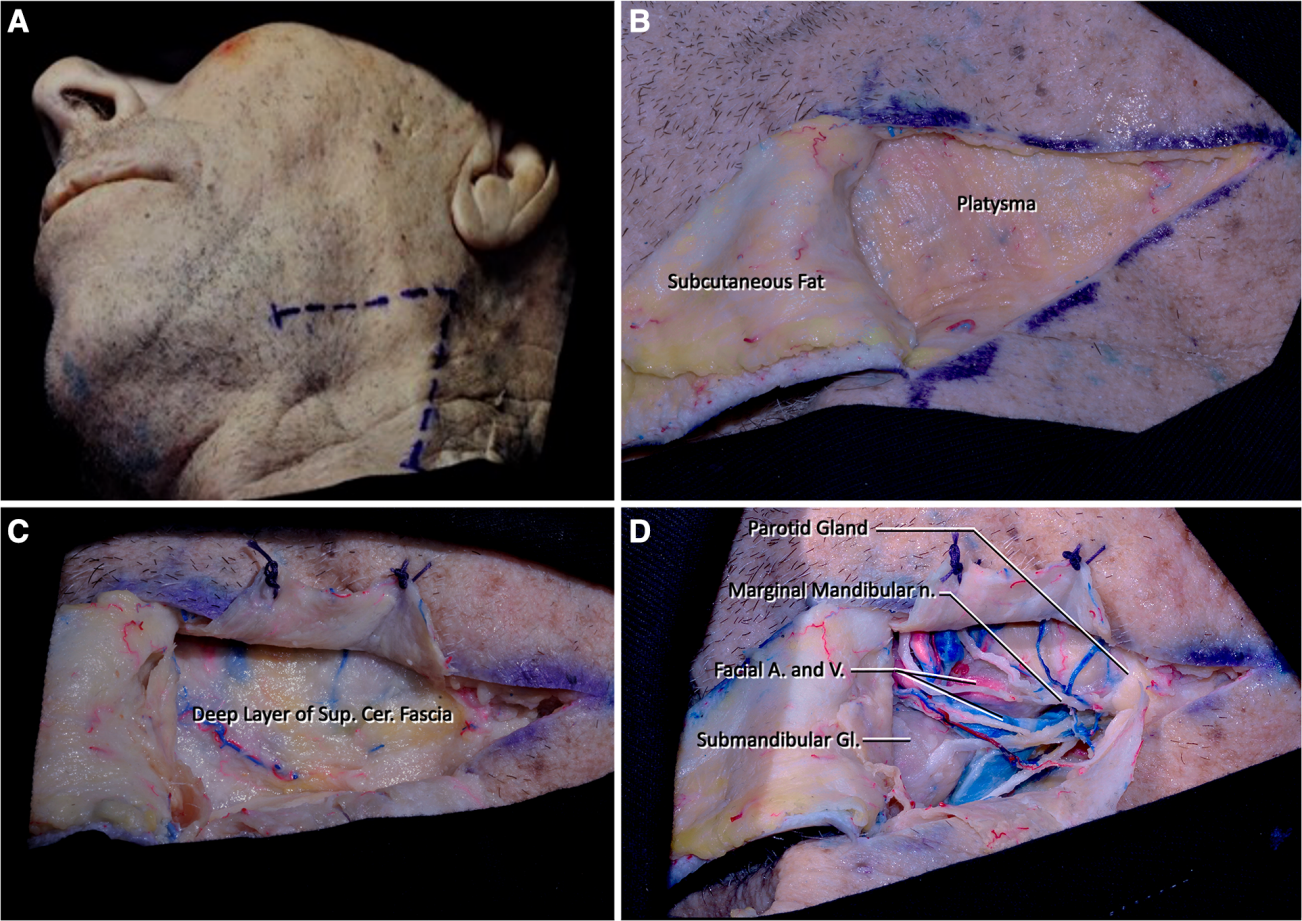
(**A**) The head is extended 15° and rotated 45° to the contralateral side. A V-shaped incision is determined by the position of the edge of the mandible and the sternocleidomastoid muscle (Step 1). (**B,C**) The skin flap is dissected, and platysma muscle is cut and elevated over its attachment. (**D**) Determining the marginal mandibular branch of the facial nerve after subplatysmal dissection. Submandibular and parotid glands should be detected at the medial and lateral sides of the dissection area, respectively. The facial artery and vein are located just lateral to the parotid gland.

Step 1. The procedure is performed with the head in Mayfield holder extended 15° and rotated 30° to the contralateral side in supine position. The edge of the mandible (gonion), inferior border of the mandible, and anterior border of the sternocleidomastoid muscle (SCM) are the important landmarks to determine the limits of skin incision. A V-shaped incision is used where the apex overlays the edge of the mandible to expose the parotid gland. The apex fell approximately 2 cm inferior and 2 cm posterior to the angle of the mandible. The horizontal leg of the V-shape continues until the submandibular gland, but the length of this incision can be shortened or lengthened according to the location of the submandibular gland; in our cadavers an average 6 cm long incision was adequate. The oblique leg of the V-shape is extended until the lower border of the posterior belly of digastric muscle (approximately 6 cm in length but must be adjusted according to the location of the posterior belly of the digastric muscle). After the V-shaped incision, the skin and superficial cervical fascia (subcutaneous tissue) are dissected and elevated toward the midline leaving the thin layer of platysma superficially. The only important structure that could be damaged during this and the following step is the marginal mandibular branch of the facial nerve (Figure 1A).

Step 2. Platysma is divided into three flaps with a Y-shaped incision. These three flaps should be elevated over the skin over their attached parts. After elevating the muscle flaps over their attachments, the superficial fascia underlying this muscle called the superficial layer of the deep cervical fascia is seen (Figures 1B,C).

Step 3. Dissection of the deep cervical fascia should be done in a similar fashion to the superficial cervical fascia. Care should be given to the dissection of this fascia as the underlying neurovascular structures can be damaged easily. At this level of dissection, fascial tissue and neural structures (i.e., marginal mandibular branch of the facial nerve) should be meticulously dissected (Figure 1D).

**Figure 2:**
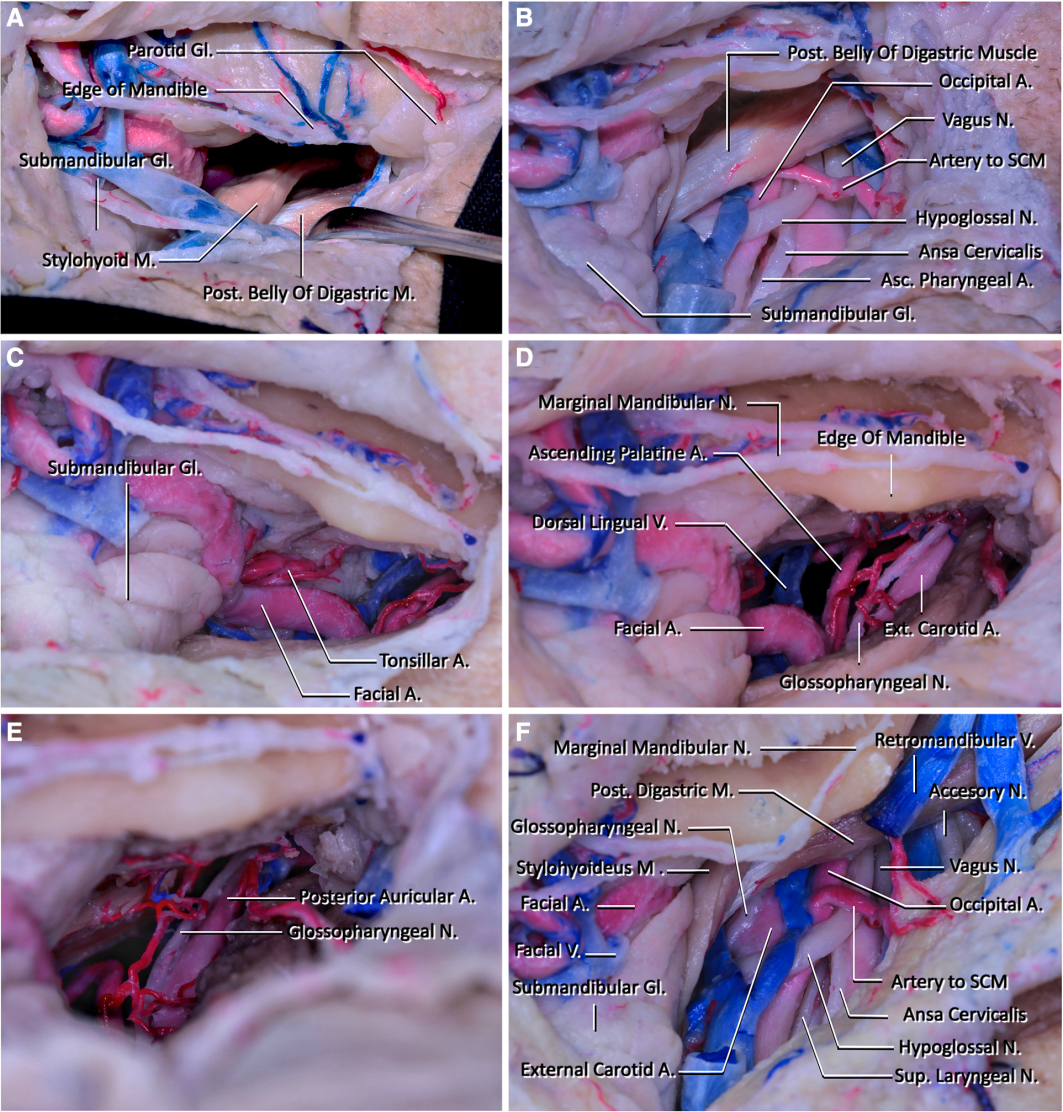
(**A**) View of the corridor that will be used to reach the clivus after subplatysmal dissection. The edge of the mandible is located superiorly, the parotid gland is located laterally, the submandibular gland and facial artery and vein are located inferiorly, the posterior belly of the digastric and stylohyoid muscles are located posteriorly, and they form the boundaries of the corridor. (**B**) The structures that are seen inferiorly and below the posterior belly of the digastric muscle after sacrificing the facial vein are demonstrated in this figure. One of the most important structures located in this region is the hypoglossal nerve. In this region, the hypoglossal nerve passes below the posterior belly of the digastric muscles and crosses internal and external carotid arteries and makes a loop. In addition, the vagus nerve coursing lateral to the internal carotid artery is seen. (**C**) The lateral view of the corridor that will be used to reach the craniocervical junction. The fourth branch of the external carotid artery, facial artery, courses at the medial wall of the retropharyngeal space and reaches superiorly just posterior to the submandibular gland and passes into the facial region. At this part, it has its characteristic tortuous shape. In this figure, the tonsillar artery is also demonstrated. It arises from the facial artery and pierces the superior pharyngeal constrictor muscle before reaching the tonsils. (**D**) The neurovascular structures that will be seen in the deep retropharyngeal space. Ascending palatine artery arising from the proximal part of the facial artery courses superiorly and crosses the styloglossus and stylopharyngeus muscles to reach the pharynx. The glossopharyngeal arises from the jugular foramen and courses inferiorly medial to the external carotid artery. It then crosses the internal carotid artery and passes below the hyoglossus muscle. (**E**) The superior wall of the retropharyngeal corridor is seen. The posterior auricular artery leaving the external carotid artery and entering the stylomastoid foramen is seen. (**F**) The retromandibular fossa's appearance after mobilizing the posterior belly of the digastric muscle and removing the parotid gland. The accessory nerve is demonstrated coursing laterally to the external jugular vein.

Step 4. Removal of the superficial layer of the deep cervical fascia reveals the parotid gland and branches of the facial nerve coming out from the parotid gland laterally. After arising between the lobes of the parotid gland, the marginal mandibular nerve (branch of facial nerve) follows a path parallel to the body of the mandible at the superior region of our surgical site. Medially, the lateral half of the submandibular gland can be seen. Just superior to the submandibular gland, facial artery arising from the external carotid artery (ECA) and the facial vein that drains into the internal jugular vein can be seen. The superior border of the incision is formed by the body of the mandible, the edge of the mandible, and the masseter muscle. An inferior border is formed by the stylohyoid muscle and the posterior belly of the digastric muscle (Figures 1D, 2A).

Step 5. Dissection of the connective tissue adjacent to the stylohyoid and posterior digastric muscles is done to enable inferior retraction of these muscles. The hypoglossal nerve (HN) underlies these muscles, thus care should be taken at this step. After the muscles are retracted inferiorly, it is possible to see the ECA, the origin of the facial artery arising from it, and HN (Figure 2B). After arising from ECA, the facial artery follows a tortuous path superomedially and gives off its first two branches, ascending palatine and tonsillar artery. After giving off these branches, it moves just superior to the submandibular gland and leaves the submandibular triangle crossing the body of the mandible (Figure 2C). The dorsal lingual vein and vena comitans of HN join at the origin point of the facial artery and form the facial vein, where it then drains into the internal jugular vein.

**Figure 3:**
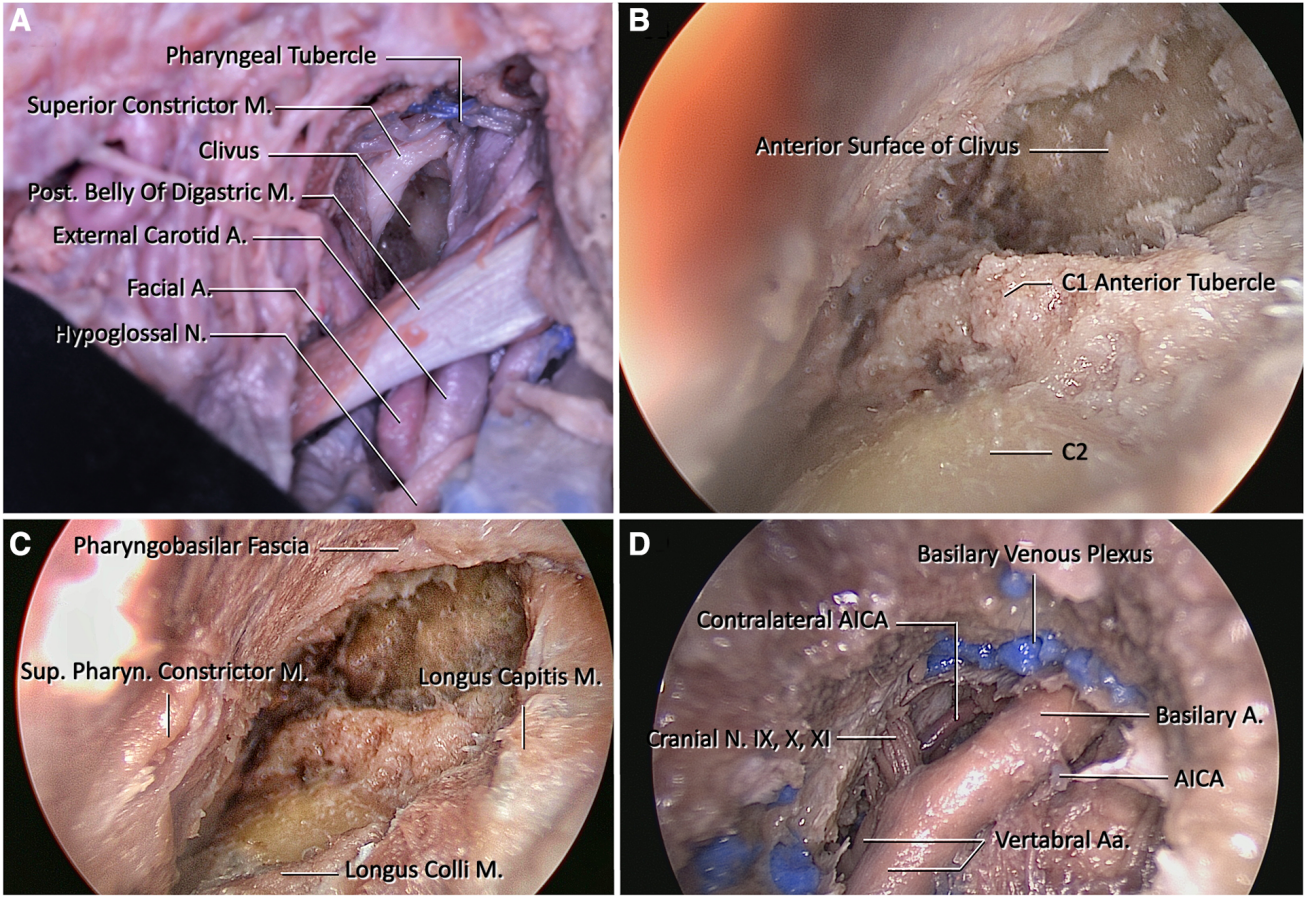
(**A**) View of the clivus after macroscopic dissection of the retropharyngeal space. (**B**) Endoscopic view of the craniovertebral junction after removal of the atlanto-occipital membrane. The anterior tubercle of C1 and the pharyngeal tubercle are important landmarks for midline orientation. (**C**) At this figure, borders of the clival regions are longus capitis laterally, and pharyngobasilar fascia superiorly. To reach the midclivus, the pharyngobasilar fascia should be dissected superiorly and exposure should be extended until the level of vomer. (**D**) View after drilling of the lower clivus and dural incision. Blue painted structures are basilar venous plexuses that are located between the dural folds. They are formed by bilateral inferior petrosal sinuses. Prior to the opening of the clival dura, the dura at this region should be cauterized. After opening the dura, the endoscope shows bilateral vertebral arteries, proximal basilar artery, bilateral anterior inferior cerebellar arteries, and cranial nerves IX, X, and XI.

Step 6. The dissection is continued deeper into the parapharyngeal space by retracting the external palatine vein, the ascending palatine artery, and the facial artery medially, and the carotid sheath laterally (Figures 2D,E). After these structures are retracted to the sides, the pharyngeal constrictor muscles are followed medially while staying under the styloglossus muscle until the longus capitis muscle is reached (Figure 3C).

Step 7. The pharynx is retracted medially using deep retractors, and after, the longus colli muscle can be seen. After the proper exposure, the pre-vertebral fascia is separated and the longus colli muscles are dissected and retracted laterally with Cloward retractors.

Step 8. In this step, the rigid 4 mm endoscope is placed in the area and the anterior arch of C1 and the anterior tubercle are exposed. The atlanto-occipital membrane, located between the C1 anterior arch and the anterior foramen magnum, is also dissected and then the ventral face of the lower clivus and middle clivus are explored (Figure 3B).

Step 9. The clivus is explored until the foramina lacera are seen on both sides. After the pharyngeal tubercle is seen and the midline is determined, clivectomy is started. Then, using a high-speed drill and a 3-mm diamond tip, clivectomy is performed up to the lower border of the sphenoid sinus at the top and laterally up to the cortical bone border sparing the foramen lacerum. A bone window of approximately 20 mm × 15 mm can be opened. And then ipsilateral C1 anterior arch is drilled to increase exposure. At this stage, the odontoid and the transverse ligament are left intact, and the dura is then opened with a vertical incision. The basilar venous plexus is located between the periosteal and meningeal leaves of the dura. After crossing this venous plexus, the ipsilateral and contralateral vertebral arteries are exposed at the inferior view converging as the BA. The anterior pontine segment of the AICA, the first branch of the BA, comprises the arterial structures that appear after clivectomy. At the lowest border of the clivectomy, at the level where the vertebral arteries form BA, the 9th, 10th, and 11th CN on both sides are visible (Figure 3D).

### 3.2 An illustrative case

A 66-year-old man presented with neck pain and numbness in his left arm. He had a history of humerus chondrosarcoma that was resected and replaced with a prosthesis 24 years ago. He had also undergone surgery for lung and hip metastases from the chondrosarcoma 10 years ago, respectively. On examination, the patient had limited shoulder movement and arm atrophy in his left arm due to the humerus prosthesis. He also had partial restriction of forearm movements, but normal wrist movements and muscle strength. He had no neurological deficits. Magnetic resonance imaging (MRI) scan revealed a large, expansile lesion involving the C3 vertebra. The lesion was invading the surrounding soft tissues and causing spinal cord compression. The patient underwent an anterior cervical approach for resection of the tumor. The tumor was successfully removed, and the patient's symptoms improved. He was then given radiation therapy. One year later, the patient developed a recurrence of the tumor. The tumor was resected again, and the C2–C5 vertebrae were fused with iliac crest autograft and an anterior screw-plate system. The patient received post-operative radiation therapy and remained disease-free for 5 years. Five years after the second surgery, the patient developed another recurrence of the tumor. The tumor was resected again, and the C2–C4 vertebrae were corpectomized and reconstructed with iliac crest autograft. The patient then underwent posterior occipitocervical fusion.

**Figure 4:**
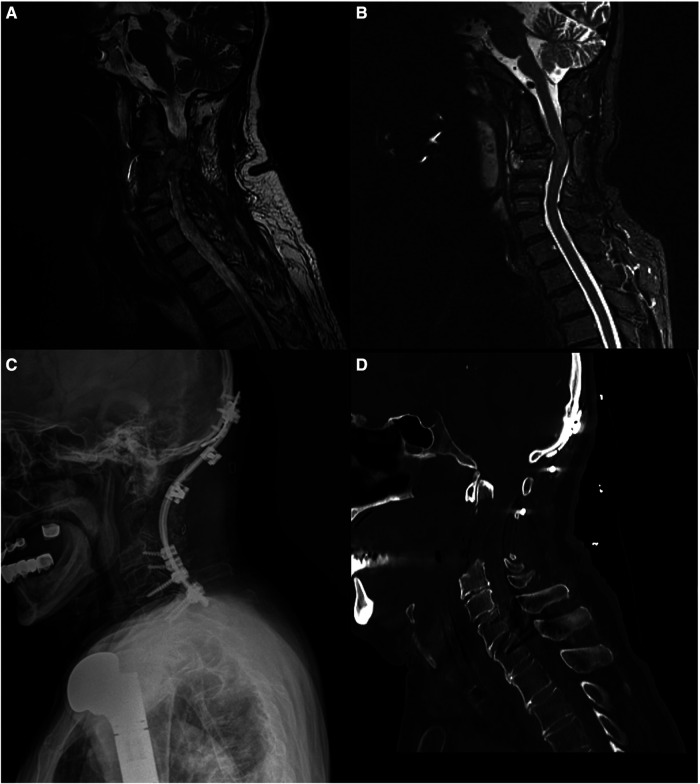
(**A**) A pre-operative MRI scan showed a lesion located in the C3 vertebra. (**B**) MRI scan of the recurrent lesion prior to the third surgery (**C**) Cervical x-ray after occipitocervical stabilization surgery. (**D**) Cervical computed tomography (CT) scan after tumor resection and copectomies of C2, C3, and C4 vertebrae.

In our case, the lesion involved the C2 vertebral body, odontoid process, and extended into the C3 and C4 vertebral bodies. Although a transoral approach could access the C2 vertebral body and odontoid process, it was deemed unsuitable due to the lesion's extension to the C3 and C4 vertebrae and the higher complication rate associated with the transoral approach. Therefore, the high cervical anterolateral retropharyngeal approach was chosen in this case for tumor resection and anterior spinal cord decompression (Figure 4).

## 4 Discussion

The High Cervical Anterolateral Retropharyngeal Approach is primarily suitable for ventral and midline lesions of the lower clivus, foramen magnum, and C1 and C2 vertebrae. The most common tumors in this region are chordomas, chondromas, meningiomas, and schwannomas (1). However, in tumors with lateral and posterior extensions, suboccipital or transcondylar approaches may be a more appropriate choice. In addition, this approach is also suitable for cases of atlantoaxial dislocation to allow for anterior decompression at the upper cervical level.

Conventionally, the first popularized approach to the lower clivus and upper cervical spine is the transoral approach. The main drawbacks of the transoral route compared with the retropharyngeal approach are the infection rates and oropalatopharyngeal complications (11, 12). In addition to the sterile surgical environment, the post-operative recovery time is significantly shorter in the anterolateral retropharyngeal transclival approach due to tracheostomy and the nasogastric tube not being needed (12). The anterior retropharyngeal approach provides an adequate exposure of the anterior superior cervical spine and lower clivus, while maintaining a wider lateral exposure. One of the disadvantages of this approach is the apparent focused exposure to the lower clivus. In the study of Agrawal et al., where the transoral and extraoral approaches were compared, the working area of the transcervical approach was wider than the transoral approach, but the working length on the C1 anterior tubercle was longer than the transoral approach. In addition, the transoral approach has an anterior-to-posterior trajectory, while the transcervical approach has a lateral-to-medial trajectory. This means that the transoral approach provides a straight view, while the transcervical has an oblique view to the C1 anterior tubercle (11).

The endoscopic endonasal approach has become a popular mode of approach to the lower clivus in recent years. Its main advantage is the wide viewing angle and access to the upper and middle clivus. Since using a natural anatomical corridor, the lack of the need for retraction makes this approach superior in certain regards (13). However, a major disadvantage is the relatively common cerebrospinal fluid (CSF) leakage. In the review study conducted by Morales-Valero et al. where 20 studies describing the endoscopic endonasal approach in craniovertebral junction pathologies were examined, 18% of the patients developed intraoperative and 4.2% post-operative CSF leaks (14). The risk of CSF leakage is also possible in the transcervical approach, sans the risk of meningitis due to the aforementioned sterile environment. As the target of the surgery shifts caudally to the lower clivus and upper cervical spine, the retropharyngeal approach takes the advantage in exposure, which allows instrumentation if needed (10). The endoscopic endonasal approach also has an anterior-to-posterior trajectory, similar to the transoral approach.

Modified transcervical approach is only indicated for lesions involving the lower and middle clivus. In this approach, clivus access can be achieved by partial resection of the ipsilateral C1 anterior arch. This allows for preservation of the atlantoaxial ligaments and consequently reduces the risk of post-operative craniocervical instability. In addition, if the target lesion extends into the upper cervical region and requires a more extensive resection, craniocervical instability is inevitable.

On the other hand, the limited space in which important neurovascular and visceral structures are located can cause difficulties for surgeons using the retropharyngeal approach. Extensive retraction may lead to injury of pharyngeal musculature, whereas limited retraction of the midline musculature may lead to limited visualization of the midline and contralateral structures.

The purposed approach of this study differs from the previously described transcervical approaches with regard to patient positioning, initial incision, used corridor, and dissection. Many of the previously described incisions include a horizontal incision starting from the tip of the mastoid coursing until the symphysis menti. Complementary vertical (T-shaped) (6, 7) or oblique (L-shaped) (5) oriented incisions have been described in the literature (3, 8). The V-shaped (modified Schobinger) incision of this study starts with a horizontal incision starting at a point just inferoposterior to the angle of the mandible. The aim here is to expose the anterior part of the parotid gland to visualize branches of the facial nerve arising at the posterior pole of the parotid gland. The incision continues parallel to the body of the mandible just 3 cm inferior to it to decrease the possibility of injuring the marginal mandibular branch of the facial nerve (8). The oblique part of the V starts from the apex, and courses parallel to the SCM muscle until it reaches the posterior belly of the digastric muscle. The oblique part of the V, parallel to SCM, is also shorter than previously described incisions (Figure 3) (11). The oblique part of the incision can be lengthened inferiorly and the corridor below the digastric muscle can be used to reach pathologies below the C2 vertebra. This corridor coincides with the angle of the mandible, which can be used as a landmark to maintain good orientation of this corridor in deeper dissection. In addition, retraction of the submandibular gland is not necessary, which decreases the risk of injuring the marginal mandibular branch of the facial nerve, lingual nerve, HN, facial artery, and vein (15–17). However, a large submandibular gland can still cause obstruction to the surgical view and exposure. Another cause for limited visualization can be a small jaw. In these cases, the submandibular gland can be dissected from the surrounding tissues and mobilized or excised. Freeing the digastric muscle from the hyoid bone by removing the fibrous sling and separating the posterior belly of the digastric muscle from the stylohyoid muscle was used previously to allow an open corridor for the surgery, but a similar exposure can be gained with gentle retraction of the posterior belly of the digastric muscle inferiorly. Limiting this step minimizes the risk of injuring the ansa cervicalis, HN, glossopharyngeal nerve, superior laryngeal nerve, superior thyroidal artery, carotid sheath, and the structures in them (18–20).

One of the most serious difficulties of the transcervical approach is the need for retraction. More severe complications can also occur including paresis of the superior laryngeal nerve and HN due to excessive retraction (21, 22). However, since the approach is submandibular rather than subdigastric, the injury risk of the superior laryngeal nerve compared with the conventional submandibular approach is lower. The superior laryngeal nerve is out of the operation field, and this minimizes the possibility of superior laryngeal nerve palsy in the post-operative period (20). It can also be argued that performing this approach with endoscope will reduce the need for retraction and therefore reduce the complication rate.

Marginal mandibular nerve injury is another complication of the transcervical approach. While dissecting the superficial layer of the deep cervical fascia, one should be extremely cautious with the marginal mandibular branch of the facial nerve. This nerve is one of the structures that has a higher injury potential. To decrease this probability, the incision is made 3–4 cm below the body of the mandible and after identifying the marginal mandibular branch of the facial nerve, it can be dissected and mobilized superiorly to avoid injury in the following steps of the dissection (23). At the same time, it is thought that using an exoscope in these macroscopic stages of the procedure will make it easier to recognize the anatomical structures and reduce the possibility of injury to these structures. In case of injury, an asymmetrical smile, and elevation of the lower lip on the ipsilateral side can be seen post-operatively (17, 24).

**Figure 5:**
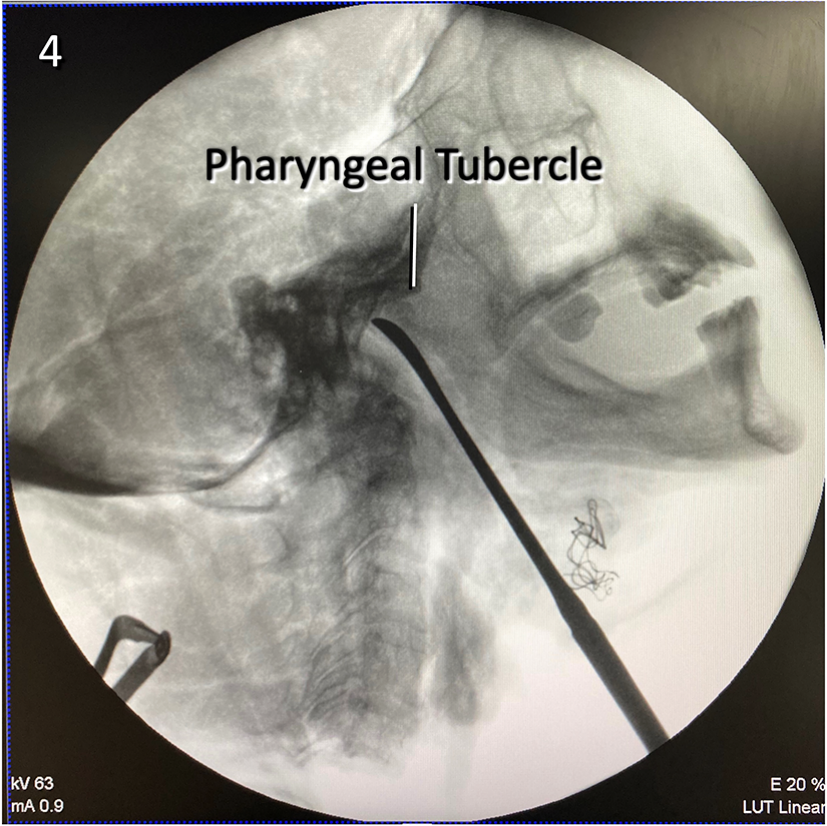
Lateral x-ray view of the retropharyngeal corridor used to reach the craniocervical junction. The tip of the dissector points to the lower clivus.

Maintaining midline orientation is vital for the safety of the deep dissection for the submandibular retropharyngeal approach. To provide adequate exposure to the surgical target, head rotation is required, which makes the midline orientation harder. This positioning may vary depending on the surgeon, but an angle of 15° extension and 30° rotation to the contralateral side is adequate (6–8, 25, 26). The angle of the mandible should be positioned to avoid obscuring the surgeon's field of view (Figure 5). Another important point is the position of the clivus. Clival drilling should not be started without proper orientation. Without proper orientation, the vertebral arteries at C1 level remain at risk while looping over the posterior arch of the C1 vertebra. At this stage, the pharyngeal tubercle or C1 anterior tubercle can be used as a landmark. Another stage where midline orientation is vital is dissection of the retropharyngeal space. Retromandibular fossa is located superolateral to the retropharyngeal space and it includes the temporal artery, the spinal accessory nerve, and the hypoglossal nerve, which necessitates care while working on the superior and lateral borders (27).

The use of an endoscope in the transcervical approach makes this approach more reliable as previously discussed in the literature (2). Since rigid retraction is not needed as much, the risk of retraction-related injuries decrease. At the same time, angled endoscopes provide a wider field of view compared with microscopes. In this way, the major shortcomings associated with the long working corridor can be overcome.

There are various but a limited number of anatomic studies in the literature describing the high cervical retropharyngeal approach in clivus and craniocervical junction pathologies (3, 8, 11, 28, 29). The study of Russo et al. is an endoscope-assisted anatomical study that uses the corridor under the digastric muscle and includes minimal anterior clivectomy (8). Baird et al. compared endoscopic endonasal, transoral, and transcervical methods in terms of working angle, distance to clivus, and working areas (3). Agrawal et al. compared transoral and transcervical approaches in terms of distance from the dura and tip of the anterior tubercle of C1 (11). Salle et al. described the endoscopic anatomical study using the submandibular corridor and measured the distances between eight predetermined points in the craniocervical region (28). Li et al. used the corridor under the digastric muscle to reach the craniocervical junction and discussed the lateral boundaries of the clivectomy. In addition, the effects of the atlas and odontoid resections on the extent of exposure have been shown (29). Although it had been shown in previous literature, our experience shows that it is impossible to view the clival region straight on similar to the endonasal approaches in the transcervical approaches because the pharyngeal muscles and the midline structures cannot be lateralized beyond the midline (8, 29). In addition, in lesions that are limited to the clivus that do not extend into the upper cervical vertebral area, using the corridor in the submandibular trigone over the digastric muscle will both reduce the working distance to the clivus and reduce the risk of neurovascular injury due to retraction.

## 5 Limitations

This study has several limitations that should be considered when interpreting the results and translating them to clinical practice. Our study focused solely on the anatomical feasibility of the modified HCALR approach using cadaveric dissections. While this provides valuable insights into the approach's potential, it does not translate directly to clinical outcomes in live patients. The study did not assess the potential complications and risks associated with the modified HCALR approach in a clinical setting. Real-world factors like bleeding, tissue manipulation, and surgical technique can significantly impact complication rates. It is recommended to use the exoscope during superficial macroscopic stages to better simulate real surgical procedures.

By addressing these limitations and conducting further research, we can gain a more comprehensive understanding of the modified HCALR approach’s potential role in clinical practice.

## 6 Conclusion

In this study, a modified high cervical anterolateral retropharyngeal transclival approach is described and compared with the methods described previously. This study is highly demonstrative of the regional anatomy, all important neurovascular structures, and their relations to each other with stepwise dissections. It could serve as a guide for other surgeons to lower complications.

## Data Availability

The raw data supporting the conclusions of this article will be made available by the authors, without undue reservation.
