# Supplementary material for: Endoscopy-assisted high cervical anterolateral retropharyngeal approach to clivus: a cadaveric study
Source: Front Surg. 2024 Jul 22;11:1397729. doi: 10.3389/fsurg.2024.1397729 (PMC11298333; doi:10.3389/fsurg.2024.1397729)
Supplement: Supplementary file 1 [file Table1.docx]

# Table 1

| STEPS | DETAILS And SUGGESTIONS | IMPORTANT STRUCTURES |
| --- | --- | --- |
| STEP 1  Determining Landmarks And  ‘V’ Shaped İncision | Apex of the incision is 2 cm posterior and lateral to angle of mandible. Horizontal leg incision was from apex until submandibular gland, oblique incision was parallel to SCM muscle. Both legs were 6 centimeters long. | Marginal mandibular branch of facial nerve |
| STEP 2  Cutting And Retraction Of The Platysma Muscle | Y-shaped cut of the platysma, 3 leaves of the muscle were retracted to sides. | Marginal mandibular branch of facial nerve |
| STEP 3  Dissection Of The Subplatysmal Tissue | Injury of the marginal mandibular branch of the facial nerve is most probable at this step. Submandibular and parotid gland, facial nerve and digastric muscle were observed after dissection of the subplatysmal muscle. | Marginal mandibular branch of facial nerve  Facial vein and artery  Submandibular Gland  Parotid Gland |
| STEP 4  Determining The Boundaries Of The Corridor To Be Entered İnto The Retropharyngeal Space | Borders of the corridor  Superior border: Mandible  Inferior border: Digastric muscle  Lateral border: Parotid Gland  Medial Border: Submandibular Gland | Marginal mandibular branch of facial nerve  Facial Artery and Vein  Submandibular Gland  Parotid Gland |
| STEP 5  Inferior Retraction Of Posterior Belly Of Digastric Muscle And Stylohyoid Muscle | Fibrous sling that fixes the digastric muscle to hyoid bone was gently retracted inferiorly without cutting to increase the area of entrance. Important neurovascular structures such as hypoglossal nerve pass just below the digastric muscle so the surgeon must be careful. | Hypoglossal Nerve  Ansa Cervicalis  Superior Laryngeal Nerve  Carotid Sheath |
| STEP 6  Deepening Of The Retropharyngeal Space With Blunt Dissection | The dissection is deepened into the parapharyngeal space by taking the dorsal lingual vein superiorly, ascending palatine artery and facial artery medially, and the carotid sheath laterally. | Glossopharyngeal Nerve  Dorsal Lingual Vein  Carotid Sheath |
| STEP 7  Medial Retraction Of The Pharynx After İdentified Of The Longus Capitis Muscle | To prevent postoperative pharyngeal edema and dysphagia excessive retraction should be avoided. | Pharyngeal Venous Plexus |
| STEP 8  Determination Of The Midline By İdentification Of The Longus Colli Muscle And The C1 Anterior Tubercle Using The Endoscope. | Sympathetic trunk and longus colli muscles course in proximity thus the surgeon must be cautious not to harm the sympathetic trunk while retracting and dissecting longus colli muscle. Vertebral artery leaves the transverse foramen at this level; thus, the surgeon must be careful not to lose the midline orientation and harm the vertebral artery. | Sympathetic Trunk  Vertebral Artery |
| STEP 9  Exposition Of The Lower Clivus And Doing Clivectomy By High-Speed Drill | During clivectomy, attention should be paid to the foramen lacerum on both sides of the clivus and the ICA passing through it, maintaining the midline orientation. Again, at this stage, injury to the basillary artery adjacent to the inner surface of the lower clivus should be avoided. | Ipsilateral vertebral artery  İCA  Basillary artery |
