# Supplementary material for: Endoscopy-assisted high cervical anterolateral retropharyngeal approach to clivus: a cadaveric study
Source: Front Surg. 2024 Jul 22;11:1397729. doi: 10.3389/fsurg.2024.1397729 (PMC11298333; doi:10.3389/fsurg.2024.1397729)
Supplement: Supplementary file 2 [file Table2.docx]

# Table 2. Evaluation of the surgical nuances, advantages, limitations, and complications of the modified high cervical anterolateral retropharyngeal approach

|  | |
| --- | --- |
| *Surgical nuances* | - V-shaped insicion 2 or 3 cm below the mandible - Identify the submandibular triangle and borders of the corridor - Inferior retraction of posterior belly of digastric muscle and cover the HN - C1 anterior tubercle should be identified and midline orientation should be ensured. |
| Advantages | - Minimally invasive - Improved cosmetic outcomes - Lower neurovascular injury risk - Potential for endoscopic assistance |
| *Limits* | - Limited exposure - Technical difficulty - Potential for postoperative complications - Patients with a medical condition in the neck, such as arthritis, vascular abnormalities, or a history of previous neck surgery |
| Complications | - Neurovascular injuries - Airway complications - Dysphagia - Chronic pain |

**Table 3.** Comparison of transcervical, transoral, and endoscopic endonasal approaches

|  | Transcervical | Transoral | Endoscopic endonasal |
| --- | --- | --- | --- |
| Advantages | - Low risk of infection - Shorter recovery time - Wider lateral exposure | - Straight view - Wider working area | - Wide viewing angle - Less retraction |
| Disadvantages | - Narrow working area - Pharyngeal retraction | - High infection risk - Oropalatopharyngeal complications | - CSF leak - High infection risk |

**Table 4.** Comparison of standard transcervical approach with modified transcervical approaches

|  | Standard transcervical | Modified transcervical |
| --- | --- | --- |
| Insicion | - T-shaped - L-shaped - U- shaped - Transvers | - Mini V-shaped |
| Corridor | - Carotid triangle located beneath the posterior belly of the digastric muscle | - Submandibular triangle located above the posterior belly of digastric muscle |
| Advantages | - Wide working area - Accessibility to the lower cervical vertebrae | - Closer working distance to the clivus and craniocervical region - Wider working angle - Lower risk of neurovascular injury - Better cosmetic results |

**Table 5.** Anatomical landmarks and main pitfalls of the modified high cervical anterolateral retropharyngeal approach

| Steps | Anatomical Lanmarks | Main Pitfalls | Maneuvers |
| --- | --- | --- | --- |
| 1 |  Angle of the marginal mandible   Submandibular gland   Sternocleidomastoid muscle (SCM) | Injury to the marginal mandibular branch of facial nerve | The incision should be made 3 cm below the mandible. |
| 2 |  Platysma muscle   Facial nerve | Injury to the facial nerve  Bleeding from the submandibular gland or facial vessels | Carefully dissect the platysma muscle, separating it from the underlying structures |
| 3 |  Submandibular gland   Parotid gland   Facial nerve   Digastric muscle |  Injury to the facial nerve, which can lead to facial paralysis   Damage to the submandibular gland or parotid gland |  Carefully dissect the subplatysmal tissue, identifying and preserving the underlying structures   Protect the facial nerve throughout the dissection to avoid injury |
| 4 |  Mandible   Digastric muscle   Parotid gland   Submandibular gland | - | Carefully identify the anatomical landmarks to define the boundaries of the corridor |
| 5 |  Digastric muscle (posterior belly)   Stylohyoid muscle   Hypoglossal nerve   Ansa cervicalis   Superior laryngeal nerve   Carotid sheath |  Injury to the hypoglossal nerve, which can lead to tongue paralysis   Damage to the ansa cervicalis or superior laryngeal nerve, causing swallowing difficulties   Injury to the carotid sheath, leading to potential vascular complications | Carefully retract the digastric muscle and stylohyoid muscle inferiorly, minimizing dissection to preserve surrounding structures |
| 6 |  Dorsal lingual vein   Ascending palatine artery   Facial artery   Carotid sheath | Injury to the dorsal lingual vein, ascending palatine artery, or facial artery, leading to bleeding | Use blunt dissection techniques to deepen the retropharyngeal space, minimizing tissue trauma |
| 7 |  Pharynx   Longus capitis muscle   Pharyngeal venous plexus |  Excessive retraction of the pharynx, potentially causing postoperative pharyngeal edema and dysphagia   Injury to the pharyngeal venous plexus, leading to bleeding |  Carefully identify the longus capitis muscle   Retract the pharynx minimally to expose the surgical field without causing |
| 8 |  Longus colli muscle   C1 anterior tubercle   Sympathetic trunk   Vertebral artery |  Injury to the sympathetic trunk   Injury to the vertebral artery   Loss of midline orientation | - |
| 9 |  Foramen lacerum   Bilateral ICA   Basilar artery   Ventral surface of the lower clivus |  Injury to the ICA   Injury to the basilar artery |  Identify the foramen lacerum on each side   Use a high-speed drill to carefully resect the clivus |
